# Cyberphysical Human Sexual Behavior Acquisition System (SeBA): Development and Implementation Study in China

**DOI:** 10.2196/12677

**Published:** 2020-04-03

**Authors:** Xiaoping Zhou, Jichao Zhao, Xun Liang

**Affiliations:** 1 Beijing Key Laboratory of Intelligent Processing for Building Big Data Beijing University of Civil Engineering and Architecture Beijing China; 2 China National Petroleum Corporation Managers Training Institute Beijing China; 3 School of Information Science Qufu Normal University Qufu China

**Keywords:** cyberphysical system, sexual behavior, smart sex toys, mobile social network

## Abstract

**Background:**

Sexual health is one of the principal components of human well-being. Traditional methods for observing human sexual behavior typically adopt manual intervention approaches (eg, interviews). However, the data obtained by such traditional approaches suffer from intrinsic bias and limited sample sizes. Sexual behavioral data that are more reflective of the actual situation can be collected by equipping sex toys with sensors.

**Objective:**

To address the limitations of traditional human sexual behavior data observation methods, a novel cyberphysical system is proposed to capture natural human sexual behavior data in China at the nationwide level.

**Methods:**

A cyberphysical human sexual behavior acquisition system (SeBA) was designed and implemented. SeBA jointly utilizes state of the art information and communication technologies such as smart sex toys, smartphones, and mobile social networks. Smart sex toys enable objective collection of data on human sexual behavior, while the mobile social network provides the possibility of partnered sex in a cyberphysical manner. The objectives and function settings are discussed, and the overall framework of the system architecture is presented.

**Results:**

Operation and privacy policies are proposed and the technical solution of SeBA is described. The effectiveness of SeBA was verified based on analysis of users’ human sexual behavior data collected from January 2016 to June 2017. A total of 103,424 solo sexual behaviors were recorded involving 13,047 users, and 61,007 partnered sexual behaviors from 7,140 users were observed. The proportions of males and females in the solo and partnered sex groups were fairly consistent with recent statistics on unmarried individuals in China. We also found that only a small portion of individuals provided information on at least one other attribute besides the required input of gender, such as age, height, location, job, sex preferences, purposes, and interests.

**Conclusions:**

To the best of our knowledge, this is the first study to analyze objective human sexual behavior data at the nationwide level. Although the data are restricted to China, this study can provide insight for further research on human sexual behavior based on the huge amount of data available from wireless smart sex toys worldwide. It is anticipated that findings from such objective big data analyses can help deepen our understanding of sexual behavior, as well as improve sexual health and sexual wellness.

## Introduction

As one of the principal components of human well-being, human sexual health has been studied extensively [1,2]. Human sexual behavior is the manner in which humans experience and express their sexuality, and is a crucial factor to measure and study human sexual health [3,4]. Due to the lack of large-scale objective human sexual behavior data, much remains to be uncovered in the field of human sexual health. Human sexual behavior acquisition is the process of measuring human sexual behavior, including both solo sex and partnered sex. Thus, the development of large-scale objective human sexual behavior acquisition systems can provide new insight into the sexual lives of people. It is anticipated that a human sexual behavior acquisition system capable of capturing real-life sexual data can advance current human sexual research.

Human sexual behavior acquisition is technically challenging. Owing to concerns of sexual privacy, few individuals are willing to be observed during their sexual acts. Therefore, current solutions for capturing sexual behavior data are based on subjective reports and interviews [5-8]. However, individuals are seldom concerned about sexual behavior during an exhilarating session of lovemaking and are therefore likely to be biased (eg, overestimating time). Moreover, the number of subjects included in interviews is generally limited. For clinical purposes, some patients may allow being inspected during their sexual acts [9-11], which could somewhat affect the mood and might not exactly reflect the natural flow of sex. These problems trigger demands for new schemes to objectively measure sexual behavior.

Here, we present a new scheme, termed cyberphysical human **Se**xual **B**ehavior **A**ctivation system (SeBA), which was designed to capture natural sexual behavior. SeBA jointly utilizes state of the art information and communication technologies, including Internet of Things (IoT), big data, smart devices, cloud computing, and mobile social networks. Although the primary objective was to collect data on objective sexual behaviors with smart sex toys at a nationwide level in China, it is also possible to obtain more data from the social perspective (eg, individuals’ sexual orientation) [12]. Ultimately, more knowledge on sexual health can be discovered by big data–driven approaches using such large-scale objective human sexual behavior data, thereby contributing to complementing theories of sexuality, improving societal knowledge of the prevalence of sexual behaviors [13], and providing benefits to clinicians working to improve sexual health [14,15].

As a natural human behavior [16], people are engaging in sexual activities at all times in all places worldwide. However, much remains to be uncovered on human sexual behavior due to the lack of objective data. People engage in a variety of sexual acts, ranging from solo sex to partnered sex in varying patterns of frequency for a wide variety of reasons. Currently, no promising solution capable of capturing large-scale objective sexual behavior data is available. Thus, we designed SeBA as a novel objective sexual behavioral data acquisition system.

Sex toys such as dildos and vibrators are primarily used as masturbation tools for a single individual to facilitate their sexual pleasure. By employing sex toys, individuals can reach orgasm naturally without possible psychological effects. In other words, the sexual behavior data from sex toys could be much more objective than those obtained from interviews. Consequently, sexual behavioral data that are more reflective of the actual situation can be collected by equipping sex toys with sensors. This is the primary motivation of the proposed SeBA. A smart device equipped with various sensors is a powerful tool to sense all types of human behaviors, and has been extensively used in a variety of areas [17]. For example, smart sex toys [18] enable the collection of users’ sexual behaviors without their awareness, thus providing sexual behavior data in a natural way. However, several challenges remain to obtain sexual data from smart sex toys.

The first and primary problem is the challenge in obtaining human-human sexual behavior data from smart sex toys. Usually, sex toys, including smart sex toys and sex robots [19], are designed for masturbation. Individuals enjoy solo sex in a private space, whereas partnered sex is remarkably different. Therefore, exploring partnered sex can reveal more insight on human sexual behavior. Consequently, breaking through the space limitation of sex toys is a crucial issue.

Second, the recorded sexual behavior data should be sent back to the data center through some form of communication technology. Although sensors are embedded in sex toys to sense accurate human sexual behavior, these data are only stored in the smart sex toys. Undoubtedly, the sex toys, as well as the sexual behavior data, are the properties of users, and third parties cannot collect these data legally. Moreover, the technology of transferring the sexual behavior data from sex toys to the data center also requires the users’ permission.

Third, it is difficult to attract nationwide individuals to participate in such studies. One of the limitations of current solutions is the limited number of samples. Therefore, recruiting more individuals can result in more accurate findings on human sexual behavior.

This study proposes a novel framework, SeBA, to address the above issues.

## Methods

### Function Settings

A smartphone is a handheld personal computer with extensive computing capabilities, including high-speed access to the internet using either wifi or a mobile broadband connection. Moreover, smartphones include support for Bluetooth connectivity. With continuing progress in smartphone and mobile network technologies, the physical distance limitation of communications among individuals can be avoided by providing a cyberspace for connectivity. Currently, mobile social networks are pervasively used in our daily lives. Therefore, we employed a mobile social network as a bridge among smart sex toys through the internet in the development of SeBA.

The mobile social network embedded in SeBA is called SNApp. SNApp can be downloaded online and installed on a smartphone, allowing smartphones to connect to sex toys through Bluetooth and to the internet through wifi or mobile broadband. Moreover, individuals can connect with others on SNApp. Once a relationship is built in the social network, the linked users can interoperate each others’ sex toys, thus enabling partnered sex through SNApp. To some extent, this kind of partnered sex can be treated as typical human-human sex.

With the advantages of SNApp, SeBA solves the three main issues mentioned above. Since the cyberspace in SNApp enables users to contact other users in a private physical space, they can profit from sexual pleasure with assurance of privacy protection. Undoubtedly, SNApp is fundamentally a tool for human-human sex. Moreover, the user behavior data, as well as sexual behavior data, can be transmitted through the mobile network to the data center with the users’ permission. Theoretically, individuals worldwide can register and enjoy sexual pleasure in the same platform. From this perspective, SeBA can enable data collection not only at a nationwide but also at a worldwide scale.

SNApp has some distinct features from a traditional mobile social network. Each node in a traditional mobile social network represents a user. However, each node in SNApp connects not only the user but also a smart sex toy. The mobile social network provides the cyberspace, while smart sex toys represent the physical aspect of SeBA. Otherwise, SeBA is a typical cyberphysical system.

**Figure 1:**
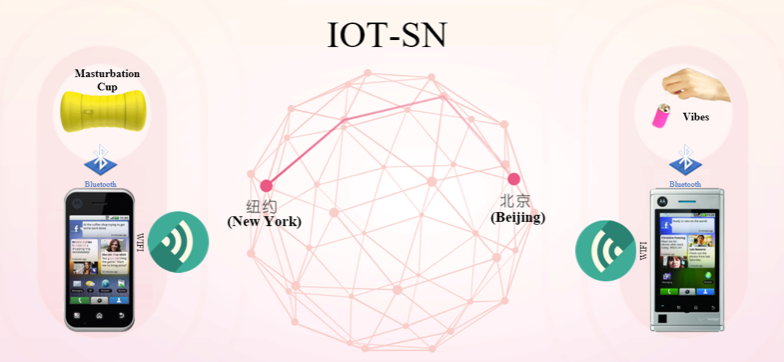
Typical usage scenario of sexual behavior acquisition system (SeBA).

Figure 1 presents the typical usage scenario of SeBA. As discussed above, users should have both smart sex toys and SNApp to enable partnered sexual behavior. Smart sex toys are devices that can be controlled via Bluetooth using SNApp. Smart sex toys can be produced in the same forms and functions as traditional sex toys with a key difference being that they include embedded chips enabling smart control from SNApp. Therefore, smart sex toys are based on the IoT concept. SNApp is the core component of SeBA. The functions of SNApp include a user function module (registration, user profile management), user-friendly function module (online user list, friend connection request/response), user communication function module (message communication, partnered sex communication), and sex toy management function module (smart sex toy connection and control). Thus, SNApp is based on a social network, which enables users to cultivate partnered sexual behavior online.

From the perspective of users, SeBA provides a platform or service to facilitate human sexual pleasure. Smart sex toys allow users to enjoy more pleasure from masturbation. Moreover, the mobile social network function enables users to find online partnered sex safely, offering almost the same feeling as penetrative intercourse. SeBA comprehensively utilizes IoT and mobile social network technologies. It is a typical cyberphysical system with the capability to sense natural human sexual behavior.

### Data Processing

To analyze the data collected from SeBA, the data were first exported and stored in a MySQL database [20]. The user statistical data were then queried using structured query language. Finally, the selected user statistical data were imported into Microsoft Excel 2016 for visualization.

## Results

### Operation and Privacy Policies

Since SeBA aims to obtain objective sexual behavioral data, the functions and operation policies focus on satisfying the users’ requirements. That is, the users pay more attention to the pleasures from the online partnered sex, with little awareness about the data problem. From this perspective, the products, including both the smart sex toys and SNApp, were designed to achieve a user-friendly experience. Moreover, unlike many other health care studies conducted by governments or hospitals, SeBA operates exclusively on the internet, which enables collecting more data from more areas.

Sex is one of the most important aspects of personal privacy. Even when an individual considers their sexual behavior to be perfectly “normal” and they have nothing to hide, they typically regard their sex lives as private. Subsequently, all of the data collected are highly private and the details of the data are encrypted to protect the leakage of users’ behavioral data.

### System Architecture

**Figure 2:**
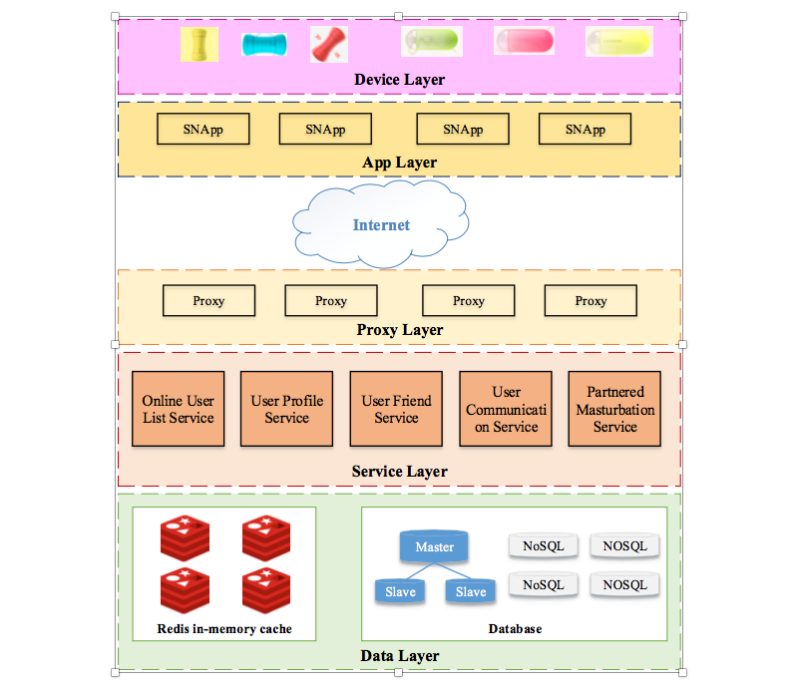
The overall system architecture of the sexual behavior acquisition (SeBA) system.

The system architecture can be roughly divided into two categories according to the users’ accessibility: local and the cloud. Figure 2 presents the overall framework of the system architecture.

The local part is composed of the hardware and software that users use. It mainly consists of the device layer and the app layer from the technical aspect. The device layer includes all types of smart sex toys, while the app layer is SNApp installed in the users’ smartphones. SNApp could be downloaded and installed from the Wolkamo Tech Co Ltd website [21].

The device layer consists of smart sex toys. Two main types of smart sex toys are produced: a smart masturbation cup and a smart vibrator. For the users, the functions of both smart toys are indistinguishable from a traditional masturbation cup and vibrator. Additionally, the smart masturbation cup and smart vibrator can be controlled by SNApp through a Bluetooth connection. Both smart sex toys are produced by Wolkamo Tech Co Ltd (Beijing, China). Since there are no international or national standards for smart sex toy, other brands of smart sex toys are not yet compatible with SeBA.

**Figure 3:**
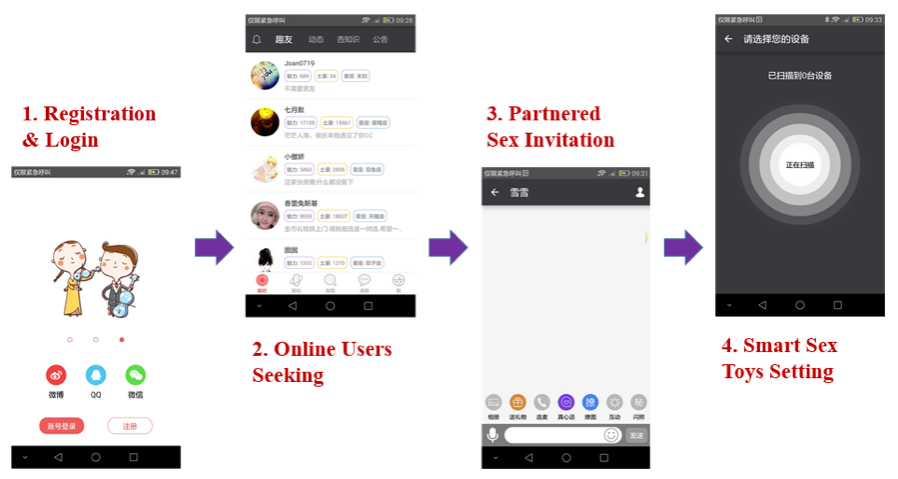
Basic usage process of sexual behavior acquisition system (SeBA).

The SNApp layer is the main user interface. SNApp can be used to control the smart masturbation cup or vibrator, and also to find a sex partner online. Users must register and then sign into SNApp to use its functions. After signing into the app, the user is marked as online and can be found by any other online users on the app. Online users can connect their smart sex toys to SNApp through Bluetooth on the smartphone. After connection, the users can start, control, and stop the sex toys in single sex or partnered sex mode by inviting another online user with a smart sex toy. In partnered sex, the two users can interact with words, pictures, and real-time voice and control their counterparts’ smart sex toys. Figure 3 presents the basic steps of SeBA.

The cloud part is the cloud services provided to support the main functions for users. It consists of three components: proxy layer, service layer, and data layer.

The proxy layer functions in three modes. First, it enables the scale-out load balance. High concurrency is the main feature of online services. The proxy layer dispatches the requests into different services in the service layer to address the high concurrency problem. Second, data compression and data encryption are provided in the proxy layer to avoid data leakage. Third, the proxy layer can act as a firewall for the cloud service, since the outer computers cannot directly connect to the computers serving the data and service layers.

The service layer provides all of the app programming interfaces for SNApp to the data layer with permissions. The main services in the service layer include an online user list service, user profile service, user friend service, user communication service, and partnered sex service. These services correlate to the function settings of SNApp.

The data layer utilizes an in-memory database to cache the real-time retrieval data, and employs a disk-based NoSQL database to store persistent data. Usually, online users are recorded in the in-memory database, since these data are frequently used to ease the users in seeking online sex partners. Currently, all other data are stored in the disk-based database.

### Effectiveness

The system was primary launched online on January 1, 2016. Up to June 8, 2017, 103,424 single sexual behaviors were recorded, involving 13,047 users, including 77.85% owning sex toys. Table 1 provides the general statistics of the users, demonstrating a greater proportion of males than females, which were completely consistent with the proportions of sex toy-owning users. With respect to partnered sex, 61,007 sexual behaviors were collected, with a slightly higher proportion of male users. We verified that these percentages were fairly consistent with recent statistics on unmarried individuals in China from the National Bureau of Statistics of China, which reported that among the 182,568 unmarried people more than 15 years of age (sampling rate 0.837‰), 107,984 (59.15%) were males and 74,584 (40.85%) were females [22].

**Table 1:** Summary of user statistics of the dataset.

User type	Total users (N)	Males (n, %)	Females (n, %)
Registered	210,104	176,904 (84.20%)	33,200 (15.80%)
Sex Toy Owners	16,760	11,037 (65.85%)	5723 (34.15%)
Solo Sex	13,047	8559 (65.60%)	4488 (34.40%)
Partnered Sex	7140	3973 (55.64%)	3167 (44.36%)

Gender is a mandatory requirement when registering an account on SeBA. SeBA explicitly reminds the user on the user registration page that their chosen gender cannot be revised after selected. It was assumed that most users will choose to be honest about their gender to find appropriate sex partners according to their preferences on SeBA.

The other attributes, including age, height, location, job, sex preferences, purposes, and interests, can be entered and revised at any time in the app setting page. Interestingly, only a small portion of individuals included details on any of these other attributes besides the required gender field. Table 2 summarizes the numbers of sex toy–owning users according to the attributes provided.

**Table 2:** Sex toy–owning users according to attribute information provided.

Attribute provided	Total sex toy users (n, % of total)	Males (n, % of total sex toy users)	Females (n, % of total sex toy users)
Height	508 (3.03%)	392 (77.2%)	116 (22.8%)
Age	485 (2.89%)	376 (77.5%)	109 (22.5%)
Location	427 (2.55%)	344 (80.6%)	83 (19.4%)
Job	261 (1.56%)	204 (78.2%)	57 (21.8%)

**Figure 4:**
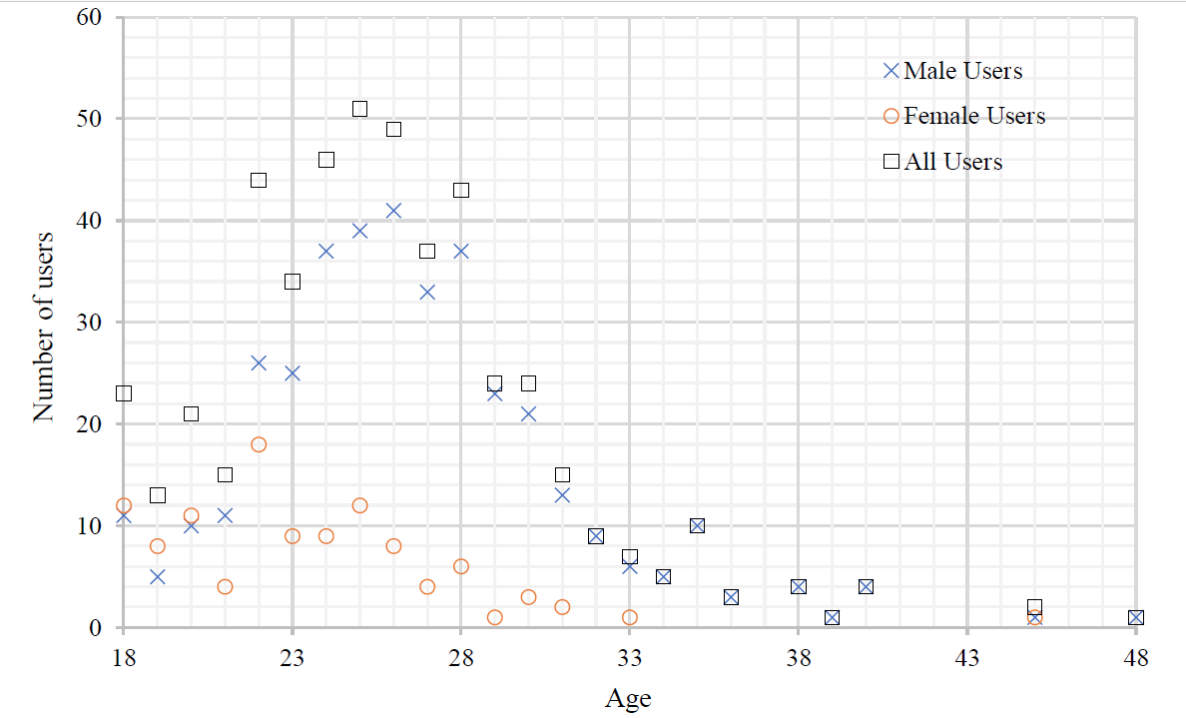
Distribution of user age in sexual behavior acquisition system (SeBA).

**Figure 5:**
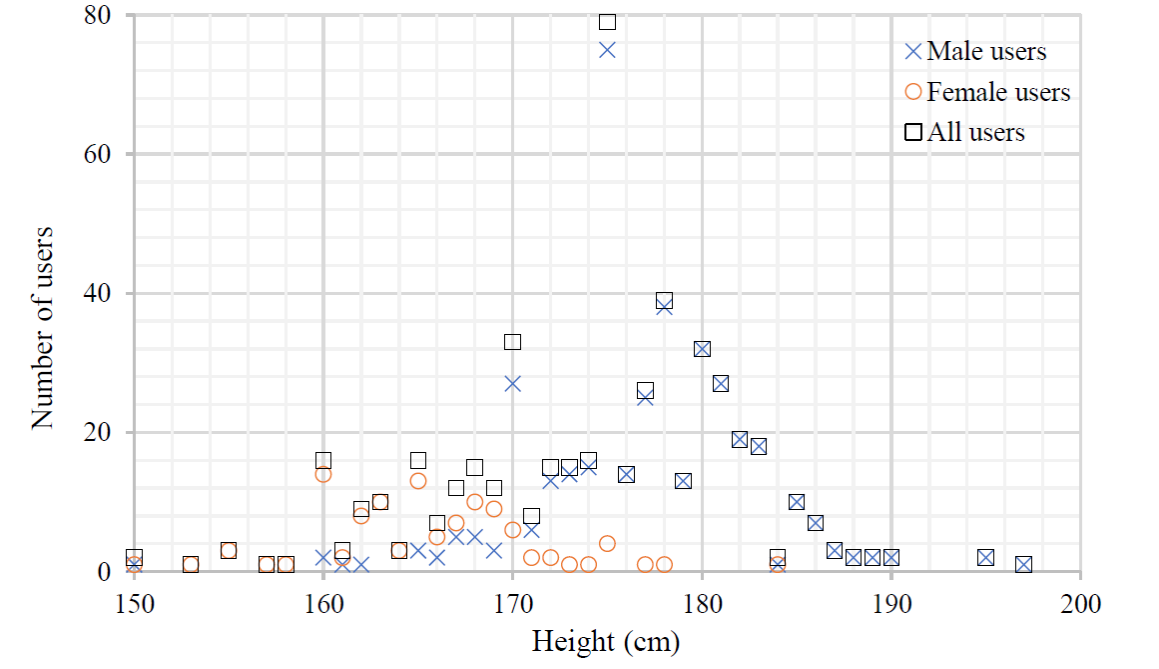
Distribution of height among sexual behavior acquisition system (SeBA).

With respect to age, most SeBA users were found to be young, with a maximum age of 30 years among the 485 users who supplied an age. Figure 4 presents the distribution of the age of these 485 users, showing a predominance of users between 22 and 28 years old. The average heights of the male and female users were 176.6 cm and 163.46 cm, respectively. Figure 5 displays the distribution of the height of the 508 users that provided this information. The height of most male users in SeBA ranged between 170 cm and 182 cm, whereas most female users reported a height between 160 cm and 170 cm. This observation is consistent with actual statistics in China [23]. Among the 427 users who entered a location, more than 20 provinces or cities in China included at least 5 individuals registered in SeBA. From this perspective, we consider that SeBA has the ability to sensor the sexual behavior data at a nationwide level. Table 3 lists the top 15 provinces or cities with the most users.

**Table 3:** Top-ranked locations of the 427 users that provided this attribute information.

Location	Male users (n)	Female users (n)	All users (n)
Beijing	56	17	73
Guangdong	49	10	59
Shanghai	25	3	28
Jiangsu	20	2	22
Zhejiang	14	3	17
Fujian	12	4	16
Hong Kong	9	5	14
Liaoning	13	1	14
Sichuan	11	1	12
Hubei	7	3	10
Hebei	9	1	10
Shanxi	9	1	10
Tianjin	9	0	9
Shaanxi	9	0	9
Macau	7	2	9

Of note, the large majority of users that provided additional attribute information were male (Table 2). Only a few females were willing to fill in additional information on their SeBA profiles. From this perspective, sexual desire plays a crucial role in online partnered sex, and most online sex behaviors are independent of age and location, but rather depend on gender selection. This finding partially confirms that users on SeBA are sexual satisfaction–oriented [24].

## Discussion

### Principal Findings

A novel cyberphysical human sexual behavior acquisition system (SeBA) was designed and implemented in China at a nationwide level. SeBA jointly utilizes state of the art information and communication technologies such as smart sex toys, smartphones, and a mobile social network. Operation and privacy policies and technical solutions of SeBA were presented, followed by preliminary data processing of collected data. The effectiveness of SeBA, in terms of the ability to collect nationwide data on human sexual behavior, was verified by more than 1 year of user data. A total of 103,424 solo sexual behaviors were recorded involving 13,047 users, and 61,007 partnered sexual behaviors from 7140 users were observed. The proportions of males and females engaging in solo and partnered sex with the app were fairly consistent with recent statistics on unmarried individuals in China. We also found that only a small portion of individuals filled in the fields of one or more other attributes besides gender, including age, height, location, job, sex preferences, purposes, and interests.

### Limitations

This is a pioneering study on collecting large-scale human sexual behavior data jointly using smart sex toys and mobile social networks. Admittedly, there are some limitations in the instrument used to collect sexual behavior. First, SeBA is currently only compatible with the smart sex toys produced by Wolkamo Tech Co Ltd due to the lack of international or national standards for smart sex toys. Second, the dataset collected may be limited to individuals who use smart sex toys and agree to share their toys on a social network. It is notable that most of the sex toys available are not yet smart. As more concerns on smart sex toys emerge, an increasing amount of sex toys are expected to be upgraded to a smart version, and thus smart sex toys from more brands will be compatible with SeBA. Third, the dataset may have failed to capture the intensities and placements of the smart sex toys in both solo and partnered sex users. Additionally, the sex that occurs via smart sex toys may not be the same as real human-human sex. With further development of smart sensors, more behavioral data can certainly be collected, resulting in more results in this area. Although the data were restricted to China, this study can provide insight for general sexual studies using a huge amount of data obtained from wireless smart sex toys worldwide.

### Conclusions

This study presents a new paradigm for human sexual behavior studies by demonstrating that smart sex toys and mobile social networks can be used to obtain objective human sexual behavior data. It is anticipated that the findings from analyzing these more objective data can help deepen our understanding of human sexual behavior, as well as help to improve sexual health and sexual wellness [25-28]. This study may contribute to demystifying the ultimate secrets of human sexuality in the era of wireless IoT and big data [29].

## Abbreviations

IoT: Internet of Things
SeBA: cyberphysical human sexual behavior acquisition system
